# Opportunistic breast cancer screening by mammography in Japan for women in their 40s at our preventive medical center: harm or benefit?

**DOI:** 10.1007/s12282-012-0367-9

**Published:** 2012-04-20

**Authors:** Mari Kikuchi, Hiroko Tsunoda, Tomomi Koyama, Toshiko Kawakita, Koyu Suzuki, Hideko Yamauchi, Osamu Takahashi, Yukihisa Saida

**Affiliations:** 1 Department of Radiology, St. Luke’s International Hospital, 9-1 Akashi-cho, Chuo-ku, Tokyo, 104-8560 Japan; 2 Center for Preventive Medicine, St. Luke’s International Hospital, Tokyo, Japan; 3 Department of Pathology, St. Luke’s International Hospital, Tokyo, Japan; 4 Breast Center, St. Luke’s International Hospital, Tokyo, Japan; 5 General Internal Medicine, St. Luke’s International Hospital, Tokyo, Japan

**Keywords:** Screening mammography, Opportunistic, Benefits and harm

## Abstract

**Background:**

After recent revised grading by the US Preventive Services Task Force of mammography (MMG) recommendations for women in their 40s, it is urgent to collect data on the benefits and harm of MMG screenings in Japan. In this paper, we study the actual status and effectiveness of opportunistic breast cancer screening by MMG for women in their 40s.

**Methods:**

From January to December 2008, the total number of opportunistic breast cancer screenings by MMG at our institute was 12823. Of them, 398 (3.1 %) who were diagnosed as category 3 or more on MMG required further exams. The data were compared between two groups (women in their 40s, women aged 50 and older). Recall rate, detection rate of breast cancers, and implementation rate of further exams were evaluated.

**Results:**

Recall rate was 4.0 % (166/4138) for women in their 40s and 2.4 % (166/6949) for women aged 50 and older. Detection rate of breast cancers was higher in women in their 40s (0.56 %) than women aged 50 and older (0.26 %). Non-cancer rate among women receiving invasive examination was higher in women in their 40s (0.76 %) than women aged 50 and older (0.42 %) (*p* = 0.02). The number of false positives required to detect one true cancer patient was smaller in women in their 40s (4.5) than women aged 50 and older (5.3).

**Conclusion:**

The results from our single institute revealed that opportunistic breast cancer screening by MMG for women in their 40s shows higher net benefits than for women aged 50 and older.

## Introduction

In November 2009, the US Preventive Services Task Force (USPSTF) changed its recommendation for MMG for women in their 40s from grade B (they had recommended screening once or twice a year for all women aged 40 and over) to grade C (the decision to undergo screening from ages 40 to 49 should be an individual one that takes into account the benefits and harm of MMG) [1]. This revision was made because MMG screening brings benefits women in their 40s to reduce 15 % mortality rate [2, 3]. However, such screening also harms (e.g., invasive biopsy or exposure to radiation as a result of further examination, a cause of anxiety, limiting of medical resources, and cost) and is considered to have little net benefit [4–9].

Accordingly, in May 2010 the Japan Association of Breast Cancer Screening (JABCS) indicated its view that “this revision of the USPSTF’s recommendations is generally appropriate in light of the scientific evidence. However, it should not be directly introduced in Japan, since the data was based on those from the USA. Breast cancer peak age in Asia may be different from that in the USA and European countries [10]. Japanese recommendations should be revised based on data specific to Japan, but Japan-specific data on harm are lacking and such data must be promptly assembled” [11]. Japan has various systems of breast cancer screening that include population-based screening as well as opportunistic screening through employer-provided medical checkups and complete medical examinations. The state of opportunistic breast cancer screening must be ascertained in order to look at data from Japan [12]. Thus, we study the actual status and effectiveness of opportunistic breast cancer screening by MMG for women in their 40s at our institute.

## Patients and methods

Opportunistic breast cancer screening by MMG was performed for a total of 12823 women from January to December 2008 at our institute, specifically 4165 for initial screenings and 8658 for subsequent screenings. Every MMG was classified into five categories according to guidelines for MMG [13]. Of them, 398 (3.1 %) who were diagnosed as category 3 or more on MMG required further exams. The data were compared between two groups (women in their 40s, women aged 50 and older).

### Evaluated subjects

Number of recall cases (rate), unidentified cases, further exams, identified outcome cases, breast cancer cases, false positive cases, positive predictive value (PPV), cancer detection rate, implementation rate of further exams, i.e., MMG, ultrasound (US), fine needle aspiration (FNA), core needle biopsy (CNB), vacuum-assisted breast biopsy (VAB), pathology, and staging of breast cancer cases were evaluated between two groups.

## Results

The age-specific results are shown in Table 1. The recall rate was higher for women in their 40s (4 %) and women aged 50 and older (2.4 %). Women who were recalled for further examination but whose subsequent status was unknown (i.e., whether or not they underwent further exam) accounted for 22.9 % of women in their 40s and 29.5 % of women aged 50 and over. Some women initially underwent further examination but failed to return when additional examination was required. This was true of one woman in her 40s and three women in their 50s. Final outcomes were accurately assessed for 127 women in their 40s (23 women found to have breast cancer, 104 false positives) and 114 women aged 50 and older (18 women found to have breast cancer, 96 false positives). The number of false negatives could not be ascertained, so the false positive rate could not be determined; thus, the number of false positives was divided by the total number of women. This rate was higher for women in their 40s (2.5 %) than for women aged 50 and older (1.4 %), but the difference was not significant. The cancer detection rate was higher for women in their 40s (0.56 %) than for women aged 50 and older (0.26 %).

**Table 1:** Age-specific results

	40–49 years	≥50 years	*p* value
Total no.	4138	6949	
No. of recall cases (rate)	166 (4.0 %) 166/4138	166 (2.4 %) 166/6949	
No. of unidentified cases (rate)	38 (22.9 %) 38/166	49 (29.5 %) 49/166	
No. of further exams (rate)	128 (77.1 %) 128/166	117 (70.5 %) 117/166	
No. of identified outcome cases	127	114	
No. of breast cancer cases	23	18	
No. of false positive cases	104	96	
False positives/total no.	2.5 % 104/4138	1.4 % 96/6949	
PPV	18.1 % 23/127	15.8 % 18/114	0.73
Cancer detection rate	0.56 % 23/4099	0.26 % 18/6897	0.00
Cancer detection rate	0.56 % 23/4138	0.26 % 18/6949	0.00

There was little difference between the total number of women seen and the number of women whose results were analyzed (number obtained by excluding women whose outcomes could not be determined from the total number of women seen), so there were no differences in the rate of cancer detection regardless of which number was used as the denominator. PPVs were derived by dividing the number of women found to have breast cancer by the number of women with known outcomes. There were no significant differences in these values for women in their 40s (18.1 %) and women aged 50 and older (15.8 %).

The implementation rate of further examination by age group is shown in Table 2. An additional MMG was undergone by almost the same rate of women in their 40s (12.5 %) as women aged 50 and older (11.1 %). An additional US was undergone by most women in their 40s (93.8 %) and most women aged 50 and older (96.6 %). US was not undergone in cases in which MMG findings were normal or with calcifications. Both CNB and VAB were undergone by 29.7 % of women in their 40s and 28.2 % of women aged 50 and over; there were no significant differences. Of women who underwent a biopsy by FNA, CNB, or VAB, those who were found to not have cancer accounted for 0.76 % of women in their 40s and 0.42 % of women aged 50 and older. Thus a significantly larger number of women in their 40s (*p* = 0.02) were found to not have cancer.

**Table 2:** Further examination results

Implementation rate of further exam	40–49 years	≥50 years	*p* value
Additional MMG	12.5	11.1	
Additional US	93.8	96.6	
FNA	12.5	12.0	
CNB	15.6	17.9	
VAB	14.0	10.3	
Biopsy implementation rate (CNB + VAB)	29.7	28.2	
Non-cancer cases among women receiving (FNA + CNB + VAB)/total number	0.76 (54–23)/4099	0.42 (47–18)/6897	0.02

The number of false positives required to detect one true cancer patient is shown in Table 3. This number was derived by dividing the number of false positives by the number of true positives (number of breast cancer cases). Fewer women in their 40s examined as false positives than did women aged 50 and older. In addition, the result of further exams was unidentified (number of recall cases − number of identified outcome cases) in 91 subjects; 39 of these women were in their 40s and 52 were aged 50 and older. Assuming a worst-case scenario in which all of these 91 women were false positives, the number of false positive cases required to detect one true cancer patient was fewer for women in their 40s (6.2) than in those aged 50 and older (8.2). Table 4 shows the pathology of breast cancer cases. The proportion of invasive carcinoma and ductal carcinoma in situ (DCIS) was similar in patients in their 40s and aged 50 and older. Table 5 shows the staging of breast cancer cases. Women aged 50 and over were more likely to have advanced cancer with lymph node metastasis.

**Table 3:** Number of false positives required to find cancer per person

Number	40–49 years	≥50 years
False positives/breast cancer cases	4.5	5.3
Worst scenario^a^	6.2	8.2
		Aged 50–59 years
USA (BCSC)	37.6	18.4
Japan (JABCS)	30.8	25.5

*BCSC* Breast Cancer Surveillance Consortium

^a^Worst scenario: the result of further exams was unidentified in 91 subjects. Worst scenario is the result if all of these 91 women are assumed to show false positive results

**Table 4:** Pathology of breast cancer cases

Pathology	40–49 years (*n* = 23)	≥50 years (*n* = 17)
Invasive carcinoma	16 (69.6 %)	12 (66.7 %)
DCIS	7 (30.4 %)	5 (27.8 %)
	(2 pure type mucinous ca.) inc.	(1 ductal carcinoma) exc.

**Table 5:** Staging of breast cancer cases

Stage	40–49 years (*n* = 22)	≥50 years (*n* = 17)
Early (DCIS + stage I)	15 (68.2 %)	10 (58.8 %)
Advanced (stage II–)	7 (31.8 %)	7 (41.2 %)
Lymph node metastasis (−)	19 (86.3 %)	10 (58.8 %)

## Discussion

JABCS has indicated that Japan cannot perfunctorily adopt the revised recommendations of the USPSTF [11]. In Japan, numerous studies have previously reported on the benefits of screening but data on its harm are lacking and must be promptly assembled. In addition, screening systems in Japan include population-based screening as well as opportunistic screening in the form of employer-provided medical checkups and complete medical examinations. The state of these types of screening must be ascertained [12]. Thus, the data on opportunistic screening at our facility were compared to population-based screening in the USA and Japan, as indicated in the literature which described screening MMG for women aged 40–49.

The results of our study were converted to a population of 1000 women undergoing screening MMG for comparison with figures from the BCSC [14, 15] and JABCS [12]. The data regarding benefits and harm of screening MMG for women in their 40s are shown in Table 6 [12, 14–16].

**Table 6:** Screening results for women aged 40–49 years

Per 1000 screened (number)	BCSC	JABCS	Our study
Harm			
False positive MMG	97.8	86.3	25.0
Additional imaging	84.3	73.4	29.3^a^
Biopsy (exclude FNA)	9.3	6.9	9.3
Benefit			
Screen-detected cancer	2.6	2.8	5.6

BCSC [12–14], JABCS: 5 prefectures [11]

^a^Additional imaging was defined as the number using US according to the JABCS

The results of our study indicated that screening MMG caused less harm in terms of both the number of false positives and the number of women who underwent an additional imaging study. This number of women who underwent an additional imaging study was defined as the number of women who underwent an US in conjunction with Japanese data on population-based screening. An additional biopsy, which did not include FNA, was the same as that of the USA. There were few differences in the number of false positives and number of women who underwent an additional biopsy, so women who are recalled for further examination at this facility are quite likely to undergo a biopsy. An improved rate of breast cancer detection is a benefit of screening MMG. The cancer detection rate in our study was higher than population-based screening in the USA and Japan.

As mentioned earlier, the number of false positives required to detect one true cancer patient was compared (bottom of Table 3). During population-based screening in the USA and Japan, more women in their 40s were false positives than women aged 50 and over, which was the opposite to our results.

Our results showed that opportunistic MMG screenings have positive net benefits. They are highly beneficial and less harmful than population-based screening in Japan or in the USA. There may be several reasons for these results. First, women in their 40s have higher breast cancer prevalence in Japan than in the USA. In the USA, breast cancer prevalence consistently rises starting in 40s and continues to late 70s. In Japan, prevalence peaks twice, once in late 40s and another in early 60s [10, 11, 17].

The second reason for these results may be due to selection bias because the subjects in this study underwent opportunistic screening. The participants of opportunistic screenings at our institute may be likely to have high health awareness and have a family history of cancer, compared to those in general population-based screenings. Moreover, selection bias may have accompanied any US screening that was conducted on the same day. This study only indicated MMG results, but subjects were also allowed to undergo US screening on the same day if they wished. Thus, young women and women with dense breast tissue on a previous MMG opted to undergo US screening. The population of this study may have been women who were diagnosed more easily by MMG.

In addition, there may be instances when local residents are screened and current images cannot be compared to previous images. Women who were seen numerous times by our facility had their current images compared to their previous images. Because examinees undergoing subsequent screenings accounted for about two-thirds of all participants at our opportunistic screening, we were able to compare current images with previous images. Thus, the proportion of cases requiring more detailed examination was lower, likely resulting in a higher rate of PPV.

Currently, an individual with a ranking of A, B, or better as stipulated by the Central Committee on Quality Control of Mammographic Screening is certified to perform and read mammograms in Japan [18]. At our facility, MMG is performed by five certified technologists and physicians with A or As ranking checked all exams. This may be the third reason for the optimal results of this study.

Nevertheless, this study had several limitations. First, the results for women who were recalled were not always ascertained, as reflected by the 22.9 % of women (38/166) who were recalled but whose subsequent status was unknown (i.e., whether or not they underwent further testing). A follow-up framework must be crafted in order to reduce the rate of women who are recalled but whose subsequent status is unknown, and this is a project for future study. A second limitation is that false negatives were not fully ascertained. However, this would require checking with the cancer registry and is a given limitation of opportunistic screening.

Encapsulating the results of screening local residents of five Japanese prefectures, the view of the JABCS is that Japan cannot perfunctorily adopt the revised recommendations of the USPSTF. Results of this study are only from one institute, but they indicate a greater net benefit for opportunistic MMG screening of women in their 40s compared to such screening of women aged 50 and older, corroborating the view of the JABCS. Currently, JABCS is involved in a nationwide survey, the results of which should be presented in the future.

## Conclusion

The results from our single institute revealed that opportunistic MMG screenings for women in their 40s show higher net benefits than for women aged 50 and older.
